# Disturbances of Tryptophan Metabolism and Risk of Depression in HCV Patients Treated with IFN-Alpha

**DOI:** 10.4172/2332-0877.1000131

**Published:** 2014-02-25

**Authors:** GF Oxenkrug, WA Turski, W Zgrajka, JV Weinstock, R Ruthazer, P Summergrad

**Affiliations:** 1 Department of Psychiatry, Tufts Medical Center/Tufts University, Boston, MA, USA; 2 Department of Experimental and Clinical Pharmacology, Medical University, Lublin, Poland; 3 Department of Toxicology, Institute of Rural Health, Lublin, Poland; 4 Division of Gastroenterology/Hepatology, Tufts Medical Center/Tufts University, Boston, MA, USA; 5 Institute for Clinical Research and Health Policy Studies, Tufts Medical Center/Tufts University, Boston, MA, USA

**Keywords:** Hepatitis C, Interferon-gamma, Depression, Tryptophan, Kynurenine, Indoleamine 2, 3-Dioxygenase

## Abstract

Depression is a common side-effect of interferon (IFN)-alpha treatment of hepatitis C virus (HCV) infection and melanoma. Disturbances of tryptophan (TRP) metabolism might contribute to development of IFN-alpha–associated depression due to IFN-alpha-induced activation of indoleamine 2,3-dioxygenase (IDO), a rate-limiting enzyme of TRP–kynurenine (KYN) metabolism. The increased frequency of high producer (T) allele of IFN-gamma (IFNG) (+874) gene, that encodes IFNG production, in depressed patients suggested that increased IDO activity might be a risk factor for depression. The present study assessed KYN/TRP ratio (KTR) as a marker of IDO activity in American Caucasian HCV patients awaiting IFN-alpha treatment. KTR did not differ between 43 patients who did and 37 patients who did not develop depression. TRP concentrations were higher in patients who experienced depression. Odds of development of depression increased with elevation of serum TRP levels from 33% (TRP levels <12000 pmol/ml) to 68% (TRP levels > 16000 pmol/ml). Elevated serum TRP may reflect the impairment of TRP conversion into serotonin in agreement with suggested link between serotonin deficiency and depression. Up-regulation of IDO might be an additional risk factor of IFN-alpha–associated depression. Future studies shall explore the causes of elevated serum TRP in relation to IFN-alpha–associated depression.

## Introduction

Depression is a common (30 – 50%) side-effect of interferon (IFN)-alpha treatment of hepatitis C virus (HCV) infection and melanoma, and might compromise the effectiveness of therapy [1]. Experimental and clinical data suggested that disturbances of tryptophan (TRP) metabolism might contribute to the development of IFN-alpha–associated depression [2]. IFN-alpha transcriptionally activates indoleamine 2,3-dioxygenase (IDO) [3], the rate-limiting enzyme of TRP conversion into kynurenine (KYN), the major (90%) non-protein pathway of TRP metabolism [4]. Up-regulation of TRP – KYN metabolism limits availability of TRP as a substrate for minor pathway of TRP metabolism, i.e, biosynthesis of serotonin and other methoxyindoles: N-acetyl serotonin and melatonin [4]. In addition to serotonin deficiency, suggested as one of the mechanisms of depression [4,5], increased formation of KYN and its neuroactive derivatives contribute to mechanisms of depression as well [6,7]. Production of IFN-gamma (IFNG), the strongest among interferons inducer of IDO, is encoded by polymorphic IFNG (+874) T/A gene [8]. We reported the association of high producer (T) allele of IFNG +874) gene with increased risk of IFN-alpha associated depression [9]. Our observation suggested the association between IDO activity and risk of depression. The present study aimed to assess IDO activity in relation to risk of IFN-alpha–associated depression in HCV patients.

## Methods

KYN/TRP ratio (KTR) was used as a marker of IDO activity [10]. Blood samples were collected after 12 hrs of fasting. Serum levels of TRP and KYN were detected by HPLC-UV-fluorometric method [11] in 80 American Caucasian HCV patients awaiting treatment by peg interferon-(IFN-) alpha (Pegasys or Peg Intron) (subcutaneous injections, 120 to 180 μg/week) in combination with ribavirin (1,000 to 1,200 mg/day). Doses were determined by the patients’ body weight. Treatment lasted for 6–12 months depending on the virus genotype. Patients were evaluated by psychiatrist, and presence/absence of depression during IFN-alpha treatment was assessed retrospectively (within 2 years after initiation of treatment) by utilization of Structured Clinical Interview for DSM-IV axis 1 Disorders (SCID) for past depression. Study was approved by Tufts Medical Center IRB, and written consents were obtained for participation in the study.

## Statistical Analysis

Quantitative data are presented using median (50-th percentile) and minimum - maximum range. Non-parametric tests (Kruskal-Wallis test and Chi-square tests) were used to assess statistical significance of the obtained data.

## Results

There were 56 males and 24 female American Caucasian HCV patients, 55.3 + 7.55 years of age. Sixty four patients had HCV genotype 1 or 4, and sixteen patients had HCV genotype 2 or 3.

Forty three patients experienced depression during IFN-alpha/ribavirin treatment. Patients who developed depression had higher TRP concentrations than patients who did not develop depression (p<0.05, Kruscal-Wallis test) (Table 1).

There were no differences in KYN concentrations and KTR between 43 patients who did and 37 patients who did not experience IFN-alpha–associated depression (Table 1).

Odds of development of depression increased as TRP levels elevated from 33% (TRP levels <12000 pmol/ml) to 68% (TRP levels>16000 pmol/ml, p<0.03) (Table 2).

## Discussion

The main finding of our study was an observation that odds of the development of IFN-alpha – associated depression were increased with elevated concentrations of serum TRP. The present results suggest that high serum TRP level might be a risk factor for the development of IFN-alpha – associated depression. Recent prospective study did not find differences in TRP serum levels between depressed (Beck Depression Inventory, BDI scores >10) and non-depressed (BDI<10) HCV patients at each time point (baseline, one and six months) during IFN-alpha treatment and 3 months post-treatment [12]. Authors did not compare baseline TRP levels in patients who develop and did not develop depression during IFN-alpha therapy. In the present retrospective study plasma TRP levels in HCV patients (not currently undergoing IFN-alpha treatment) were higher in patients who experienced than in those who did not experience depression during past IFN-alpha treatment.

TRP is an initial substrate of serotonin (and other methoxyindoles, i.e., N-acetyl serotonin and melatonin) biosynthesis [13]. Serotonin deficiency was suggested as one of the major mechanisms of depression [4, 5]. Availability of TRP as a substrate for serotonin biosynthesis is one of the rate-limiting factors of serotonin formation from TRP [14]. TRP is transported into brain by a competitive carrier system it shares with such other large neutral amino acids such as tyrosine, phenylalanine, leucine, isoleucine, and valine [14]. Elevated serum TRP concentrations might, therefore, reflect the impaired TRP transport via blood-brain-barrier (BBB). Increased serum ratio of large amino acids to TRP, suggesting impaired TRP transport via BBB, was reported in depressed patients [15]. Therefore, present observation of association of elevated concentrations of serum TRP with the odds of development of IFN-alpha – associated depression is in agreement with the serotonin deficiency hypothesis of depression.

The other rate-limiting factor of serotonin biosynthesis from TRP is the activity of TRPhydroxylase 2 (Tph2), an enzyme catalyzing TRP conversion into serotonin [16]. Clinical and experimental studies suggested the association of depression with Tph2 deficiency [17]. There may be a possibility that elevated serum TRP concentrations are associated with Tph2 deficiency.

We previously reported an association between high producer (T) allele of IFNG (+874) T/A gene with the risk of IFN-alpha – associated depression [9]. Since IFNG (+874) gene encodes the production of IFNG, the strongest inducer of IDO, we suggested that IDO activity is a risk factor for IFN-alpha – associated depression. Contrary to our suggestion, we did not find the difference between KTR (as a marker of IDO activity) in patients who develop and who did not develop IFN-alpha – associated depression. Interpretation of the present results should consider that presence of high producer (T) allele of polymorphic IFNG (+874) gene relates to the possible rate of IFNG production in response to inflammation, and might enable carriers of T allele to produce more IFNG, and, consequently, more KYN from TRP in response to IFN-alpha treatment. However, the studied patients were free from IFN-alpha/ribavirin treatment at the time of blood samples collection. In addition, activation of IDO by IFNG depends on other factors, including polymorphism of IDO gene [18]. KTR characterizes the actual serum concentrations of KYN formed from TRP as a result of IFNG-induced activation of IDO.

Up-regulation of TRP – KYN metabolism was observed in major depressive disorder [19]. However, some studies indicated that it might occur independently from TRP depletion in depressed patients [20]. Despite many similarities between IFN-alpha–associated depression and major depressive disorder [2], the exposure to chronic viral infection might modify mechanisms of depression development in HCV patients.

Our data warrant the further studies (in a larger population sample) of the relationship between TRP – serotonin and TRP – KYN pathways of TRP metabolism as risk factors for depression.

## Conclusions

Taken together, the results of our previously published [9] and present studies suggest that deficiency of serotonin formation from TRP is a major risk factor for developing of IFN-alpha – associated depression. The presence of high (T) producer allele of IFNG(+874) gene that encodes the production of IFNG, the strongest IDO inducer, might augment the risk of IFN-alpha – associated depression, especially in patients with deficient serotonin formation of TRP, by additional decrease of TRP availability as a substrate for serotonin biosynthesis.

## Figures and Tables

**Table 1:** Kynurenines and risk of IFN-alpha – associated depression.

	Depressed (n=43)	Non Depressed (n=37)	P - value (Kruskal-Wallis test)
Tryptophan: Mean ± sd# Median <q1–q3>	15405 ± 3817.4 * 14500 < 12500–17900>	13646 ± 3634.7 * 13100 < 10600–15900>	0.05
Kynurenine: Mean ± sd Median <q1–q3>	1124.8 ± 464.8 * 1020 < 860–1330>	1049.7 ± 334.8 * 970 < 880–1180>	0.62
Kynurenine/tryptophan (X100): Mean ± sd Median <q1–q3>	7.4 ± 2.6 6.9 < 5.65–9.2>	7.9 ± 2.5 7.6 < 5.9–9.36>	0.38

# sd - standard deviation;

* pmol/ml

**Table 2:** Tryptophan level and risk of IFN-alpha – associated depression.

Tryptophan level (pmol/ml)	% of depressed patients,(number of observations, depressed/total)
<=12000	33 % (6/18)
12001 –14000	52% (11/21)
14001 – 16000	54% (7/13)
16001 – high	68% (19/28)

Odds Ratio (95% CI) for depression is 1.14 (CI: 1.00–1.30) (per 1000 units); Chi-square = 4.44 (df = 1), p<0.03
